# Role of MRI-Based Radiomics in Sinonasal Cancer Management: A Scoping Review

**DOI:** 10.3390/cancers17203313

**Published:** 2025-10-14

**Authors:** Andrea Migliorelli, Marianna Manuelli, Andrea Ciorba, Francesco Stomeo, Stefano Pelucchi, Chiara Bianchini

**Affiliations:** ENT & Audiology Unit, Department of Neurosciences, University Hospital of Ferrara, 44100 Ferrara, Italy

**Keywords:** sinonasal cancer, sinonasal tumor, artificial intelligence, radiomics, machine learning, deep learning

## Abstract

**Background/Objectives:** Malignant tumors of the sinonasal tract are rare, accounting for approximately 3–5% of all head and neck tumors. Despite recent advances in therapy, overall survival in sinonasal cancer remains limited, with local recurrence representing the leading cause of treatment failure. Consequently, the preoperative identification of patients at higher risk of recurrence or developing more aggressive tumors is of critical importance to guide treatment strategies and improve outcomes. The aim of this review is to analyze the results of the most recent literature studying the use of radiomics in malignant sinonasal tumors. **Methods:** A comprehensive literature review was conducted using the PubMed/MEDLINE, EMBASE and Cochrane Library databases, in accordance with the PRISMA review criteria (from 2020 to July 2025). **Results:** The final analysis comprised a total of five articles and 629 patients. At present, radiomics in sinonasal cancer is mainly applied to the prediction of Ki-67 expression, the early assessment of recurrence risk, and the evaluation of response to induction chemotherapy. **Conclusions:** The results of this review indicate that radiomics has the potential to play an important role in the management of sinonasal malignant tumors. However, further research is necessary to confirm these findings.

## Simple Summary

Radiomics could improve the management of sinonasal cancer. In this paper, the potential applications of radiomics in the management of sinonasal cancer have been evaluated, with a particular focus on its role in the early identification of patients with more aggressive disease or at increased risk of recurrence. Whilst the results are encouraging, further validation is required before its adoption in clinical practice.

## 1. Introduction

Malignant cancer of the sinonasal tract represent a heterogeneous and rare group of tumors, accounting for approximately 3–5% of all malignant neoplasms of the head and neck [1]. The incidence of this malignancy is estimated as less than 1 case per 100,000 individuals [2]. Among these cases, sinonasal squamous cell carcinoma is the most frequent histotype, accounting for approximately 38% of cases [3,4]. These histotypes are distinguished by their aggressive biological behavior and unfavorable prognoses, primarily due to the tendency to be diagnosed at advanced stages. This is attributable to the initially asymptomatic nature or non-specific clinical symptoms, which delay diagnosis until the invasion of adjacent structures [5,6].

From a therapeutic standpoint, surgery is considered as the primary treatment option. In instances where the disease is locally advanced or presents with high-risk features, adjuvant radiotherapy is frequently employed as a complementary modality [7]. Chemotherapy is also indicated in selected, more chemosensitive histotypes, and in recent years, induction chemotherapy (IC) has been studied as a possible strategy to improve outcomes [8,9]. Despite advances in surgical techniques, radiotherapy delivery modalities and the development of multimodal approaches, overall, 5-year survival rates remain modest, ranging from 20 to 57% [3,10]. Local recurrences represent the primary cause of treatment failure, often linked to the difficulty of achieving radical resections in a complex anatomical area in close proximity to critical structures [11,12]. In this context, it is crucial to identify patients at high risk of local recurrence at the time of diagnosis.

In recent years, radiomics has been established as a novel research field with considerable potential in the domain of head and neck disorders [13,14,15,16]. Radiomics is a field of study that involves the extraction of hundreds of quantitative features from medical images [17,18,19]. This process provides information that is not directly visible in the medical imaging. The quantitative data, which may reflect intratumoral heterogeneity and correlate with biological and molecular aspects of the neoplasm, designate radiomics as a form of “virtual biopsy” of the entire tumor.

Recent studies have analyzed the role of radiomics in the diagnosis of sinonasal tumors, however available data on the management of these cancers are still scant [20,21]. The aim of this review is to analyze the use of radiomics in the management and follow-up of sinonasal cancer.

## 2. Materials and Methods

**Figure 1:**
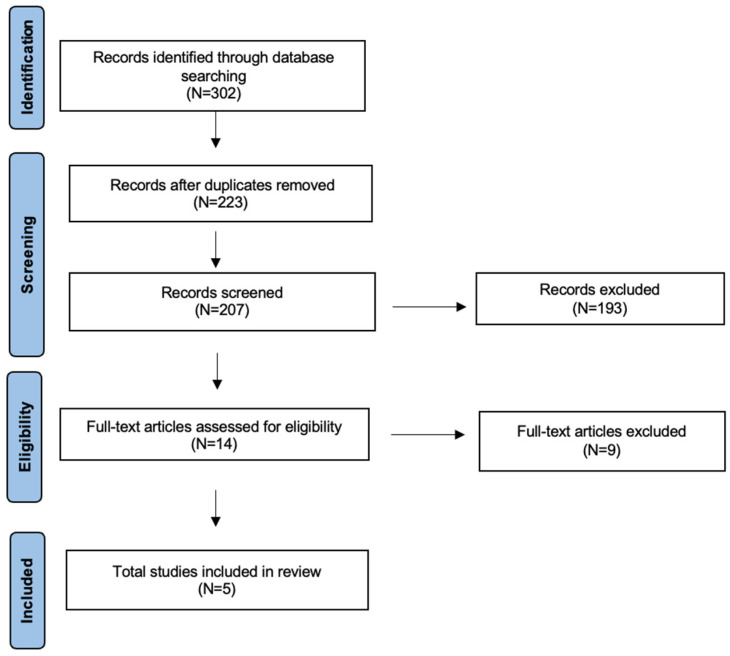
The literature review performed using PRISMA guidelines for scoping review.

A detailed review of the English-language literature on radiomics in sinonasal cancer was performed using PubMed/MEDLINE, EMBASE, and Cochrane Library databases. The search period was from 2020 to July 2025, with the aim of selecting the most recent studies. The terms used were “sinonasal cancer”, “sinonasal tumor”, “paranasal cancer”, “paranasal tumor” or “skull base cancer”, “skull base tumor” and “Deep Learning”, “DL”, “Machine Learning”, “ML” or “Radiomics”. The search yielded 302 candidate articles. The search was performed according to the “Preferred Reporting Items for Systematic Reviews and Meta-Analyses” (PRISMA) (Supplementary Materials) for scoping review guidelines (Figure 1) [22]. The inclusion criteria applied were: (i) publication date after 2020; (ii) studies aimed at using radiomics in the management or follow-up of sinonasal cancer; and (iii) English language. Conference abstracts, case reports, publications written in a language different from English have been excluded. We also excluded studies that aimed to distinguish between benign and malignant tumors of the sinonasal region, as well as those that aimed to evaluate the potential malignant progression of inverted papilloma. Two authors (AM and MM) have evaluated independently all titles, and relevant articles have been selected according to inclusion/exclusion criteria; a senior author (AC) resolved any disagreements. At the end of the full-text review, 5 articles met the inclusion criteria [23,24,25,26,27].

## 3. Results

This scoping review has included 5 articles in total, for a total of 629 patients analyzed [23,24,25,26,27]. Analyzed studies and the major findings are summarized in Table 1. The articles analyzed originate from Asia and Europe. This review shows that two studies evaluate Ki-67 expression, one evaluates the response to induction chemotherapy, and one evaluates local failure. The remaining study evaluates early recurrence. All studies analyzed used magnetic resonance imaging (MRI).

The first studies to evaluate the role of radiomics in managing sinonasal carcinomas focused on preoperative assessment of Ki-67 overexpression [23,26].

In 2021, Bi et al. [23] investigated whether radiomic signatures on MRI could be used for this purpose, using a sample of 128 patients (75 for training and 51 for validation). Preoperative imaging included pre- and post-contrast enhancement T1-weighted sections (T1-w and T1c, respectively) and fat-suppressed T2 images (FS-T2WI). Examinations were performed using 1.5 T and 3.0 T machines. Sinonasal tumors were categorized as having either high (>50%) or low (≤50%) Ki-67 expression. Following LASSO (Least Absolute Shrinkage and Selection Operator) regression, 15 features were identified and analyzed based on multiparametric MRI using both single and multiple combined parameters (combining T1-w, FS-T2WI and T1c features). The latter combined parameter achieved an area under the curve (AUC) of 0.852 and an accuracy of 86.3% in the validation group [23].

In 2024, Lin et al. [26] elaborated an integrated machine learning (ML)-based model with the same objective, which evaluated both deep learning (DL) and traditional handcrafted features (HC) based on multiparametric MRI images. The authors analyzed 231 patients with sinonasal carcinoma (185 in the training group and 46 in the testing group), evaluated by a 3 T MRI scan. FS-T2WI and T1c images were acquired for these scans, as well as diffusion-weighted imaging (DWI). The apparent diffusion coefficient (ADC) map was derived from the DWI. Following LASSO regression, 52 features (42 radiomic HC and 10 DL) were identified. The authors found that, in the test cohort, the integrated system’s AUC values reached 0.817 [26].

A few years earlier, in 2022, Lin et al. [25] analyzed the role of radiomics in predicting the risk of early recurrence in advanced sinonasal carcinomas. They developed a radiomic nomogram based on the radiomic signature and preoperative clinical features. A total of 152 patients with advanced (Stage III–IV) carcinoma who underwent a 3 T MRI scan were considered. The training group consisted of 106 patients and the external validation group of 46. DWI images were also considered, and ADC derived from this imaging. A total of 768 features were identified and reduced to eight after LASSO regression. The radiomic nomogram, which incorporated both the radiomic signature and clinical factors, demonstrated significant efficacy in identifying early recurrence, achieving an AUC of 0.92 in both the training and validation sets. These results were confirmed by Kaplan–Meier survival analysis [25].

In 2024, Park et al. [27] examined the potential of radiomics to predict the early (within 12 months) recurrence of squamous cell carcinoma of the sinuses without lymph node involvement. They analyzed 68 patients (47 in the training set and 21 in the validation set) who had undergone a 3 T MRI scan, extracting radiomic features from T2-weighted (T2-w) and T1c images. These features were then used to create two prediction models: one based purely on radiomic features, and one combining radiomic and clinical features. Prior to model construction, feature selection was performed using LASSO, RFE (recursive feature elimination) or RFE + LASSO. Both models were more effective at identifying early recurrence than the purely clinical model, with respective AUCs of 0.838 and 0.850 for the pure radiomic and combined models. No significant differences were found between the two models [27].

Finally, the only European study analyzed evaluated the role of early delta radiomics (the first early assessment performed three weeks after the start of therapy) in predicting response to IC [24]. Fifty patients were included in the study (40 in the training group and 10 in the validation group). T1-w and T2-w sequences, as well as ADC maps, were considered, and the response was evaluated using RECIST (Response Evaluation Criteria in Solid Tumors) criteria. Baseline MRI scans and scans after three weeks were compared. A total of 536 radiomic features were evaluated and the difference between the baseline MRI and the first MRI after three weeks was analyzed for each feature. Radiomic signatures were then developed for each sequence (T1w, T2w, ADC: 17, 16 and 9, respectively). The authors found monomodal AUC values of 0.70 for T1-w, 0.76 for T2-w and 0.93 for ADC. Radiological prediction of the RECIST criteria achieved 78% accuracy [24].

## 4. Discussion

In this scoping review, the role of radiomics in the management of sinonasal malignancies was analyzed. The studies reviewed primarily originate from Asia, with the number of patients divided into a training sample and an internal or external validation sample. It is evident that all studies are based on MRI, with the majority of these studies utilizing 3T images [23,24,25,26,27].

The results of our review demonstrate that MRI-based radiomics for the management of patients with sinonasal malignancies has yielded highly compelling outcomes, with the majority of studies exhibiting AUC values greater than 0.80 and frequently reaching values of 0.90 and above [23,24,25,26,27]. MRI is currently one of the most widely used imaging techniques in the study of sinonasal tumors, due to its ability to provide detailed characterization of soft tissues. Therefore, it is significant in the management of these diseases. However, despite the implementation of technological and scientific advances, the prognosis for patients diagnosed with sinonasal carcinoma remains poor, with frequent recurrences being a significant challenge [3,10,11,12]. Consequently, it is imperative to identify patients who are at high risk of recurrence at the time of diagnosis.

The advent of radiomics in recent years has given rise to several innovative scenarios in the study of medical images [17]. This discipline could offer the evaluation of digital images through a set of quantitative parameters, obtained by mathematical algorithms, offering describing the features of a lesion with an objective and reproducible tool [17]. In the domain of head and neck oncology, radiomics can be promising, either in the diagnostic process, either in the therapeutic management and patients follow-up [13,20,21,28,29].

An early application of radiomics in the sinonasal field was to prevent the malignant transformation of inverted papilloma. In 2025, Waters et al. [20] performed a systematic review on this topic, including five articles. The conclusions reached by the authors highlighted the potential of radiomics as a predictive tool for the malignant transformation of sinonasal inverted papilloma, serving as a non-invasive method to support clinical decision-making [20]. In the same year, Gravante et al. [21] evaluated the role of radiomics in discriminating between sinonasal tumors, reporting that all the studies analyzed had an AUC greater than 0.800, reaching an AUC > 0.89 and an accuracy > 0.81 when clinical-radiological variables were incorporated [21].

The present review focuses on sinonasal malignancies and their management, and it indicates that radiomics can assist clinicians in pre-treatment estimation of Ki-67, in the identification of patients at risk of early recurrence, and in the prediction of response to induction chemotherapy [23,24,25,26,27].

Ki-67 protein expression is widely recognized as an independent prognostic indicator in numerous malignant neoplasms, as it reflects cell proliferation and, consequently, the biological aggressiveness of the tumor [30,31]. High Ki-67 values have been demonstrated to be associated with more advanced stages of disease, a greater tendency to relapse and a less favorable prognosis. In sinonasal carcinomas, several studies have demonstrated that expression levels exceeding 50% are associated with reduced 5-year disease-free survival, accompanied by an elevated risk of local recurrence and distant metastasis [32,33]. Consequently, the 50% threshold has been adopted as the optimal prognostic cut-off in this setting with great frequency. In clinical practice, the assessment of Ki-67 status is commonly performed through immunohistochemistry on biopsy samples. However, this approach is subject to inherent limitations, namely the potential for invasiveness, the impact of limited sample size, and the inability to assess, in full, the biological heterogeneity of the tumor [32]. This has resulted in a requirement for non-invasive, accurate and reproducible methods that can provide a reliable preoperative estimate of the proliferation index.

In this context, radiomics has attracted a growing interest. This technology utilizes the extraction of quantitative and high-dimensional features from routine medical images [17,34,35]. This facilitates the acquisition of information that is invisible to the human eye and potentially related to biological parameters such as cellular proliferation. Several studies conducted in other oncological domains have demonstrated that the application of radiomics to advanced MRI sequences can yield substantial predictive capabilities regarding Ki-67 status, as evidenced by research in the context of gastrointestinal and breast cancer [36,37] . In the context of sinonasal tumors, Bi et al. [23] demonstrated that an MRI-based multiparametric radiomic signature exhibited the capacity to predict Ki-67 expression with an AUC of 0.852 and an accuracy of 86.3%. Subsequent research by Lin et al. [26] utilized an integrated systems approach and yielded results with an AUC of 0.817. The results of this study suggest that radiomics could represent an innovative strategy for the non-invasive assessment of Ki-67 status in sinonasal tumors. This approach would overcome the limitations of biopsy sampling and integrate the information provided by immunohistochemistry. Nevertheless, the extant literature is limited in number and marked by considerable methodological heterogeneity, which engenders difficulties in comparison.

Another interesting area of application for radiomics is the prediction of recurrence risk. It is evident that, to date, TNM staging remains a principal prognostic factor, thus guiding therapeutic decisions in patients diagnosed with sinonasal carcinomas [38]. Nevertheless, given the high degree of biological heterogeneity, divergent clinical outcomes may arise even among patients exhibiting analogous stages of the disease. Consequently, the identification of more reliable prognostic markers is crucial, particularly for the early prediction of the risk of recurrence in advanced cases. In this context, the research conducted by Lin et al. [25] yielded noteworthy findings. By examining patients in advanced stages (III-IV), the study demonstrated the potential of a radiomic nomogram, integrating clinical and radiomic data, to serve as a crucial tool in identifying patients at risk of early recurrence, achieving an AUC of 0.92. In a similar investigation, Park et al. [27] obtained results that, while slightly lower, still achieved an AUC greater than 0.800. The findings of this study underscore the efficacy of developing a radiomic nomogram that incorporates the radiomic signature with clinical characteristics, thereby facilitating enhanced precision and accuracy in results.

Furthermore, for patients in advanced stages, the management and treatment of these tumors is challenging; therefore, several protocols have suggested that a multimodal approach may improve overall patient survival [7]. As with other head and neck tumors, induction chemotherapy has been evaluated as a strategy to promote organ preservation, even in cases of locally advanced tumors of the paranasal sinus and nasal cavity [8,9]. The response to IC treatment has been shown to be a significant prognostic factor for survival, indicating that the selection of patients eligible for this approach may be of critical importance [8,9]. Nevertheless, the ability to predict the response to this type of treatment remains limited, and the scientific literature has focused on identifying possible predictive markers of response.

From a clinical perspective, the ability to predict response to IC would be of strategic value: firstly, it would maximize efficacy in patients who demonstrate early benefit; secondly, it would avoid unnecessary toxicity in patients who are not likely to respond to treatment. Therefore, IC could represent a more targeted instrument within multimodal treatment, with potential benefits in terms of both prognosis and quality of life. In 2022, Corino et al. [24] evaluated the potential of radiomics as a predictive marker of response to IC. The radiomic delta was considered and evaluated on pre-treatment images and after the first cycle of IC (3 weeks). The authors demonstrated how radiomics can play an important role in the early assessment of response to IC, achieving an AUC of 0.930 and a prediction accuracy of 78% according to RECIST criteria [24]. Although RECIST criteria are widely used as a standardized reference for tumor response evaluation, they may not fully reflect the biological behavior of the disease. In this context, long-term clinical follow-up or pathological confirmation could provide a more reliable reference standard for validating predictive models.

The most recurrent and predictive radiomics features identified across the reviewed studies were those describing tumor heterogeneity, particularly the texture-based parameters derived from Gray-Level Co-occurrence Matrix (GLCM) and Gray-Level Run-Length Matrix (GLRLM) analyses [23,24,25,26,27].

These features consistently captured subtle variations in voxel intensity and spatial organization within the tumor, reflecting the biological heterogeneity associated with proliferation, aggressiveness and treatment response.

Additionally, several studies identified first-order histogram features, such as mean intensity, kurtosis, and entropy, as well as wavelet-transformed features, as significant predictors. This suggests that both intensity distribution and multiscale texture information meaningfully contribute to model performance.

Overall, radiomics features that quantify intertumoral heterogeneity, especially those related to entropy and textural complexity, emerged as the most robust and reproducible biomarkers across the included studies.

Furthermore, several of the included studies demonstrated that integrating radiomic features with clinical variables enhanced predictive performance relative to radiomics-only models. These integrated approaches use complementary information: radiomics capture subtle imaging heterogeneity, while clinical data reflect tumor biology, stage and treatment context. In head and neck cancer, such combined models enhance both accuracy and clinical relevance as they more accurately reflect the multifactorial nature of the disease. Studies have reported higher AUC values for combined clinical–radiomic nomograms, supporting their role in improving personalized treatment planning and outcome prediction [25,27].

Radiomics represents a significant component in the advancement of precision medicine, effecting a transformation in imaging from a mere descriptive instrument to a non-invasive biomarker. The quantitative parameters it provides reflect cellular and molecular processes that are not visible to the human eye, thus offering new opportunities for personalized care. Nevertheless, methodological discrepancies between studies and the complexity of the workflow limit its routine clinical use. Moreover, MRI-based radiomics is particularly sensitive to acquisition variability; differences in scanner type, field strength and imaging protocols may substantially influence feature robustness and model performance. The lack of standardized protocols has the effect of limiting the reproducibility and generalizability of models, and as a result, radiomics is currently limited to the field of research. In the future, the development of standardized radiomic models integrated with clinical data and through the implementation of ML and DL will be essential for providing patients with increasingly accurate and personalized treatment.

Main drawbacks of this study are: (i) the limited number of studies currently available in the literature, resulting in a limited scope for this review, (ii) the relatively small number of patients included in the analyzed studies, (iii) retrospective studies and (iv) the heterogeneity of the radiomics models used, making comparisons difficult.

## 5. Conclusions

The current review highlights the role of radiomics as an emerging tool in the management of sinonasal carcinomas. At present, the applications of this technology are primarily concentrated on three domains: (i) prediction of Ki-67 expression, (ii) early estimation of recurrence risk, and (iii) early evaluation of potential response to induction chemotherapy. The available studies report encouraging results, with AUC values consistently above 0.800, yet further validation is required to confirm these findings.

However, these results should be evaluated with caution, as they are based on a limited number of studies and relatively small patient cohorts. Further, larger, multicenter investigations are necessary to validate and generalize these preliminary outcomes.

In our opinion, in the future, the integration of artificial intelligence and the development of standardized protocols will be crucial to improve comparability across studies and to enable the routine clinical application of radiomics in this clinical field.

## Figures and Tables

**Table 1:** Literature review.

Author (Yrs)	Country	Histology	Modality	N° Training/N° Validation	AI Algorithm	Objective	Major Results
Bi (2021) [23]	China	SCC 61 Other 67	MRI	77/51	LASSO regression + mRMR for feature selection; LR for final model	To assess the ability of MRI-based multiparametric radiomics to predict preoperative Ki-67 status in sinonasal malignancies.	Radiomic combined model from multiparametric MRI (T1W, FS-T2WI, T1c) achieved AUC 0.852 and accuracy 86.3%.
Corino (2022) [24]	Italy	SCC 12 SNUC 23 Other 15	MRI	40/10	SVM with features reduced via semi-supervised PCA	Delta radiomics to predict IC response in sinonasal cancer.	AUC of monomodal signatures: T1w = 0.79, T2w = 0.76, ADC = 0.93. RECIST prediction accuracy = 0.78.
Lin (2022) [25]	China	SCC	MRI	106/46	LASSO logistic regression + multivariable logistic regression for nomogram construction	Develop a nomogram integrating radiomic and clinical features to preoperatively predict early recurrence risk in advanced sinonasal squamous cell carcinoma	The nomogram demonstrated an AUC = 0.92.
Lin (2024) [26]	China	SCC	MRI	185/46	Deep transfer learning (ResNet50) for feature extraction combined with SVM, LightGBM, and ExtraTrees classifiers	Develop and validate a multiparametric MRI model combining hand-crafted and deep transfer learning features to predict Ki-67 status in squamous cell carcinoma of the nose.	RS-DL showed the best predictive ability in training and the highest AUC in testing (0.817), though not significantly better than RS-HC.
Park (2024) [27]	Republic of Korea	SCC	MRI	47/21	LASSO + RFE for feature selection; ML classifier	Assess whether MRI radiomics can predict early local failure in sinonasal squamous cell carcinoma.	In the test group, radiomic and combined models outperformed the clinical model (AUC 0.838 and 0.850 vs. 0.438), with no significant difference between them.

Legend: YRS: years, N: numbers, MRI: magnetic resonance imaging, w: weight, FS-T2WI: fat-suppressed T2 images, T1c: T1-weighted sections post contrast enhancement, ML: machine learning, DL: deep learning, AUC: area under the curve, ADC: apparent diffusion coefficient, IC: induction chemotherapy, RECIST: Response Evaluation Criteria in Solid Tumors, RS: radiomics signature, HC: handcrafted features; AI: Artificial Intelligence, LASSO: Least Absolute Shrinkage and Selection Operator, mRMR: maximum relevance minimum redundancy, LR: logistic regression, SVM: Support Vector Machine, PCA: principal component analysis, RFE: recursive feature elimination, SCC: squamous cell cancer; SNUC: sinonasal undifferentiated cancer.

## Data Availability

No new data were created or analyzed in this study.

## Supplementary Materials

The following supporting information can be downloaded at: https://www.mdpi.com/article/10.3390/cancers17203313/s1, Table S1. Preferred Reporting Items for Systematic reviews and Meta-Analyses extension for Scoping Reviews (PRISMA-ScR) Checklist.
